# Neo-Salvarsan and General Paralysis

**Published:** 1914-06

**Authors:** 


**Neo-Salvarsan and General Paralysis.** J. Nin Posades, La Semana Medica, December 4, 1913, reports the case of a lawyer, 40 years old, married, who had syphilis of fifteen years standing that had been treated regularly by means of mercurial inunctions. Two years ago vertigo and digestive symptoms set in, also loss of memory, disturbances of speech, difficulty in writing, zones of anaesthesia appeared and epileptiform attacks. The patient lost all desire to work and appeared absolutely indifferent to his surroundings. He still retained an interest in the matter of dress for he filled his wardrobes with clothing and refused to wear any one article of apparel more than two or three times. But when he commenced attending the theater without having previously consulted his family, Dr. Posadas was called. He found tremor of the tongue and hands, diminution of the reflexes and cutaneous sensibility, unequal pupils and slow response and the Romberg pronounced. The patient was at times hysterical and at others indifferent. 3 grammes 30 centigrammes of neo-salvarsan were administered in all, in eight doses. The first two doses of 0.30 grammes—the remainder of 0.45 grammes, with the result that all symptoms save the inequality of the pupils disappeared. One year afterward, four additional injections ot 0.45 grammes were given to make sure. The author does not state how the drug was administered, whether by intramuscular or intravenous route.

